# Impact of a Pharmacist-Managed Outpatient Parenteral Antimicrobial Therapy (OPAT) Service on Cost Savings and Clinical Outcomes at an Academic Medical Center

**DOI:** 10.1017/ash.2022.374

**Published:** 2023-01-17

**Authors:** Taylor M. Epperson, Kiya K. Bennett, Katherine K. Kupiec, Kathy Speigel, Stephen B. Neely, Beth H. Resman-Targoff, Karen K. Kinney, Bryan P. White

**Affiliations:** 1 Department of Pharmacy, Parkland Health, Dallas, Texas; 2 Clinical and Administrative Sciences, Department of Pharmacy, University of Oklahoma College of Pharmacy, Oklahoma City, Oklahoma; 3 Department of Pharmacy, University of Oklahoma Medical Center, Oklahoma City, Oklahoma; 4 Department of Nursing, University of Oklahoma Medical Center, Oklahoma City, Oklahoma; 5 Infectious Diseases Section, Department of Internal Medicine, University of Oklahoma College of Medicine, Oklahoma City, Oklahoma

## Abstract

**Background:**

Outpatient antimicrobial therapy (OPAT) is managed by a variety of teams, but primarily through an infectious disease clinic. At our medical center, OPAT monitoring is performed telephonically by pharmacists through a collaborative practice agreement under the supervision of an infectious disease physician. The effect of telephonic monitoring of OPAT by pharmacists on patient outcomes is unknown.

**Methods:**

This retrospective cohort study was conducted between July 2017 and July 2018 at a 350-bed academic medical center and included adult patients discharged home on IV antibiotics or oral linezolid. The experimental group comprised patients discharged with a consultation for the OPAT management program, whereas the control group comprised patients discharged home without a consultation. The primary outcome was 30-day readmission.

**Results:**

In total, 399 patients were included: 243 patients in the OPAT management program group and 156 patients in the control group. The 30-day readmission rates were similar in each cohort (20% vs 19%; *P* = .8193); however, the 30-day readmission rates were lower in the OPAT management program for patients discharged on vancomycin (19.4% vs 39.1%; *P* = .004).

**Conclusions:**

We did not find a difference in 30-day readmissions between patients receiving pharmacy-driven OPAT management services and those who did not. Patients receiving vancomycin via OPAT had lower 30-day readmissions when included in the pharmacist-driven OPAT management program. Institutions with limited resources may consider reserving OPAT management services for patients receiving antimicrobials that require pharmacokinetic dosing and/or close monitoring.

Outpatient parenteral antimicrobial therapy (OPAT) is the administration of parenteral antibiotics outside the hospital setting.^1^ OPAT use has been associated with decreased hospital length of stay and costs while increasing patient satisfaction.^2,3^ Previous studies have noted 30-day readmission rates ranging from 8% to 26% in patients receiving OPAT.^4–6^

The structure of OPAT programs varies widely. A 2012 survey of US infectious disease (ID) physicians showed that 44% did not have a formal OPAT program but rather utilized office staff for day-to-day follow-up.^11^ Most OPAT programs are managed by an ID clinic and have shown decreased readmissions with the implementation of follow-up visits.^12,13^ Pharmacist involvement in OPAT programs has been shown to improve monitoring,^14^ appropriate patient selection,^15^ and drug choice. Additionally, pharmacists are able to assist with adverse-event management.^9^ Based on these observations, pharmacist involvement is recommended by the 2019 UK good practice guidelines for OPAT.^17^

To reduce hospital readmissions and extensive hospital lengths of stay, the Infectious Diseases Section of the Department of Internal Medicine at the University of Oklahoma Medical Center (OUMC) entered into a collaborative practice agreement (CPA) with the Department of Pharmacy to create an OPAT service in July of 2016. This OPAT management service is unique in that patients are solely contacted via telephone. The program involves monitoring of both intravenous (IV) and oral antimicrobials, and day-to-day services of the program are primarily pharmacist driven. The service collaborates with home infusion and home health companies. Through this CPA, a clinical pharmacy specialist is authorized to provide outpatient antimicrobial management services for patients referred by hospital providers to the home antimicrobial monitoring service. The pharmacist participates in a variety of services, including evaluation of the safety and appropriateness of the antimicrobial therapy upon initial consultation placement, titration and adjustment of antimicrobial dosing regimens with guidance from various hospital protocols (eg, vancomycin, aminoglycoside, automatic renal adjustment, IV to PO interchange), ordering necessary laboratory tests to serially assess safety of antimicrobial medications, monitoring adherence to the antimicrobial therapy regimen via drug concentrations, coordination with home-infusion and home health companies, and ensuring that appropriate end-of-therapy measures are completed. The OUMC OPAT service receives requests for 300–400 consultations per year.

Although previous studies have demonstrated the benefit of pharmacy involvement in OPAT programs, no studies have directly compared outcomes between patients receiving care through a pharmacist-managed OPAT monitoring program and those who are not.^14,15,18–20^ We compared hospital length of stay and readmission rates for patients discharged with a pharmacist-driven OPAT service at an academic medical center versus those who were not discharged with the program.

## Methods

This single-center, retrospective analysis was conducted at a 350-bed academic medical center between July 2017 and July 2018. The experimental group comprised patients discharged home with an order for OPAT monitoring, and the control group comprised patients discharged home without an OPAT monitoring order. Included patients were aged ≥18 years and were discharged home with IV antimicrobial therapy or oral linezolid. Previously enrolled patients were excluded from the study. Patients were screened for enrollment in the study via a log obtained from the peripherally inserted central catheter (PICC) consultation team.

The primary objective of this study was all-cause readmission within 30 days of discharge. Secondary objectives included infection-related readmission within 30 days of discharge, all-cause and infection-related readmission within 3 days of discharge, hospital length of stay during index admission and readmission, and infection-related emergency department (ED) visits within 60 days of discharge. Infection-related readmission was defined as readmission due to recurrence or worsening of the initial infection or development of a secondary infection. The total duration of OPAT therapy was defined as the total planned OPAT duration starting the day after discharge from the hospital. A post hoc analysis was performed assessing 30-day readmission for patients receiving vancomycin. Cost savings for this analysis was calculated using costs of 30-day readmissions.

A pilot study and medication use evaluation (MUE) was conducted in August 2018 to compare length of stay and readmission rates between patients discharged on IV antimicrobials with and without orders for the OPAT monitoring program. Based on preliminary results from 1 month of data collection, which favored the OPAT monitoring program cohort, the decision was made to transition this project from an MUE to a research study. Utilizing 30-day readmission rates from the MUE of 15.6% (OPAT monitoring program) and 24.0% (OPAT without monitoring program), a power analysis was conducted using G*Power version 3.1.9.2 software to determine the necessary sample size for a directional test using power of 80% and an α of 0.05. Assuming group sizes to be equal, 299 patients were needed in each group (total n=598).

Descriptive statistics and inferential statistics were utilized to summarize and compare demographic and clinical characteristics. Frequency and percentage were used when data were categorical. Asymptotic Pearson χ^2^ tests were used to determine association between nominal and categorical data. If data were sparse, exact Pearson χ^2^ or Fisher exact tests were used. Shapiro-Wilk tests were used to determine the normality of data. Medians (interquartile range) were used to describe continuous data that failed normality tests. For nonnormally distributed variables, Mann-Whitney and Kruskal-Wallis tests were used. Multiple variable logistic regression was used to determine the odds (95% confidence interval) of 30-day readmission considering demographic and clinical characteristics. Forward selection was used to determine inclusion in the final model. All *P* values were 2-sided, and a *P* < .05. was considered statistically significant. This project was approved by the local institutional review board.

## Results

Of the 3,538 patients screened, 399 patients met inclusion criteria: 243 patients in the OPAT monitoring program and 156 patients in the control group. In total, 3,106 patients were excluded because they did not meet inclusion criteria or had already been included in the study. Baseline demographics are summarized in Table 1. Patients were predominantly white adults aged in their mid-50s, and patients most commonly had either private insurance, Medicare or Medicaid, or no insurance. Notably, half of the included patients had a previous hospital admission in the previous 12 months, and a large portion of patients had various comorbid diseases, most commonly diabetes (33%) or malignancy (26%). There were significantly more patients in the OPAT monitoring program with bone/joint infections (29% vs 15%; *P* = .001) and more patients in the control group with pneumonia (3% vs 8%; *P* = .0276). Rates of other infection types were similar between groups. We detected statistically significant differences for the number of infection sites, with most patients having only 1 primary site of infection but more patients in the OPAT monitoring program having 2 sites of infection (*P* = .041). A statistically significantly higher number of patients in the OPAT monitoring program received vancomycin (38% vs 15%; *P* < .001). Most patients were treated with monotherapy; however, a larger percentage of patients in the OPAT monitoring program were treated with dual therapy (24% vs 13%; *P* = .022). Lastly, the total duration of OPAT therapy was longer in the OPAT monitoring program, with a median duration of 32 days versus 14 days in the control group (*P* < .001).

**Table 1.** Baseline Characteristics

Characteristics	Overall (n=399)	OPAT (n=243)	Control (n=156)	*P* Value
Age, median y [IQR]	54 [43–64]	53 [44–63]	54 [41–65]	.7926
Sex, male, no. (%)	212 (53)	132 (54)	80 (51)	.5528
Race, white, no. (%)	294 (74)	186 (77)	108 (69)	.1055
Body mass index, median kg/m^2^ [IQR]	27.2 [23.8–33.4] n=364	27.2 [23.9–33.8] n=221	27.2 [23.6–32.0] n=143	.5994
Serum creatinine, median mg/dL [IQR]	0.80 [0.62–1.07]	0.79 [0.62–1.04]	0.81 [0.61–1.22]	.1175
Creatinine clearance, median mL/min [IQR]	95.5 [67–125] n=366	98.5 [68–124] n=222	89 [59–126] n=144	.3214
**Insurance status, no. (%)**				.7282
Private	112 (28)	72 (30)	40 (26)	
Medicare	102 (26)	64 (26)	38 (24)	
Medicaid	71 (18)	39 (16)	32 (21)	
Dual Medicare/Medicaid	35 (9)	22 (9)	13 (8)	
None	79 (20)	46 (19)	33 (21)	
**Past medical history, no. (%)**				
Admission in last 12 months	201 (50)	124 (51)	77 (49)	.7637
Diabetes mellitus	130 (33)	78 (32)	52 (33)	.7636
Heart failure	24 (6)	11 (5)	13 (8)	.1197
Chronic kidney disease	42 (11)	18 (7)	24 (15)	.0105
Intravenous drug use (IVDU)	12 (3)	6 (2)	6 (4)	.5500
Malignancy	102 (26)	57 (23)	45 (29)	.2229
Solid tumor	77 (75)	44 (77)	33 (73)	…
Liquid tumor	25 (25)	13 (23)	12 (27)	…
**Most common primary services upon discharge, no. (%)**				.0481
Hospitalist	141 (35)	85 (35)	56 (36)	
Internal medicine	74 (19)	44 (18)	30 (19)	
Orthopedics	37 (9)	30 (12)	7 (4)	
**Site of infection, no. (%)**				
Bone/joint	93 (23)	70 (29)	23 (15)	.0012
Bone/joint + hardware	37 (9)	27 (11)	10 (6)	.1142
Central nervous system	22 (6)	17 (7)	5 (3)	.1055
Bacteremia	132 (33)	75 (31)	57 (37)	.2398
Skin/skin structure	115 (29)	76 (31)	39 (25)	.1768
Urinary tract	53 (13)	30 (12)	23 (15)	.4910
Pneumonia	19 (5)	7 (3)	12 (8)	.0276
Intra-abdominal	27 (7)	14 (6)	13 (8)	.3182
**No. of infection sites, no. (%)**				.0409
1	259 (65)	146 (60)	113 (72)	
2	131 (33)	91 (37)	40 (26)	
3	9 (2)	6 (2)	3 (2)	
**Causative organism, no. (%)**				
MRSA	61 (15)	41 (17)	20 (13)	.2724
MSSA	64 (16)	38 (16)	26 (17)	.7847
CoNS	63 (16)	40 (16)	23 (15)	.6462
*Escherichia coli*	59 (15)	33 (14)	26 (17)	.3967
*Klebsiella* spp	25 (6)	16 (7)	9 (6)	.7430
*Pseudomonas*	24 (6)	13 (5)	11 (7)	.4855
**Organisms isolated, no. (%)**				.8207
0	4 (1)	2 (1)	2 (1)	
1	272 (68)	168 (69)	104 (67)	
≥2	123 (31)	73 (30)	50 (32)	
Multidrug–resistant organism (MDRO) isolated	136 (34)	82 (34)	54 (35)	.8450
**Common antimicrobial agents, no. (%)**				
Vancomycin	116 (29)	93 (38)	23 (15)	<.001
Ceftriaxone	68 (17)	35 (14)	33 (21)	.0801
Piperacillin-tazobactam	60 (15)	32 (13)	28 (18)	.1924
Cefazolin	40 (10)	20 (8)	20 (13)	.1363
Linezolid	41 (10)	18 (7.4)	23 (15)	.0185
Cefepime	26 (7)	17 (7)	9 (6)	.6281
Antibiotics in OPAT regimen, no. (%)				.0224
1	314 (79)	182 (75)	132 (85)	
2	80 (20)	59 (24)	21 (13)	
3	5 (1)	2 (1)	3 (2)	
Total OPAT duration, median d [IQR]	24 [11–37]	32 [12–38]	14 [8–27]	<.001

Note. OPAT, outpatient parenteral antimicrobial therapy; IQR, interquartile range; MRSA, methicillin-resistant *Staphylococcus aureus*; MSSA, methicillin-sensitive *Staphylococcus aureus*; CoNS, coagulase-negative *Staphylococcus*.

Regarding the primary outcome, there was no difference between the OPAT monitoring program and control group for 30-day readmission rate (20% vs 19%; *P* = .82) (Table 2). Reasons for readmission varied and were categorized as being related to the index infection or other reasons. Both the initial admission length of stay and the readmission length of stay were 1 day shorter in the OPAT monitoring program, though the difference was not statistically significant. The rate of infection-related readmission within 30 days of discharge was also similar between groups (*P* = .73). Rates of infection-related ED visits within 60 days of discharge were 7% in the OPAT monitoring program and 8% in the control group (*P* = .54), and collective rates of infection-related and all-cause readmissions within 3 days of discharge were 0% and 2%, respectively.

**Table 2.** Primary and Secondary Outcome Analyses

Outcome	OPAT (n=243)	Control (n=156)	*P* Value
**Primary outcome**			
30-day readmission rate, no. (%)	49 (20)	30 (19)	.8193
**Reason for readmission, no. (%)^a^**			
PICC/midline complication	4 (8)	0 (0)	
Antibiotic-related ADR	10 (20)	1 (3)	
Related to index infection	13 (27)	12 (40)	
Related to secondary infection	6 (12)	3 (10)	
Other	17 (35)	18 (60)	
**Secondary outcomes**			
Hospital LOS, median d [IQR]	7 [5–11]	8 [4–11]	.5937
Readmission LOS, median d [IQR]	5 [3–10]	6 [3–11]	.6410
30-d infection-related readmission, no. (%)	20 (8)	13 (8)	.7289
Infection-related ED visits within 60 d of discharge, no. (%)	16 (7)	13 (8)	.5406
3-d all-cause readmission, no. (%)	4 (2)	3 (2)	1.000
3-d infection-related readmission, no. (%)	0 (0)	0 (0)	N/A

Note. PICC, peripherally inserted central catheter; ADR, adverse drug reaction; LOS, length of stay; ED, emergency department; OPAT, outpatient parenteral antimicrobial therapy; IQR, interquartile range.

a Not mutually exclusive.

A post hoc, stratified analysis was performed for all patients who were discharged on vancomycin, and it showed significantly lower rates of readmission for patients in the OPAT monitoring program (19.4% vs 39.1%; *P* = .004) (Table 3). Each vancomycin readmission translated into an average cost of $18,872 and a cost savings of $339,690, assuming that the patients receiving vancomycin via the OPAT management program would save the same readmissions as the control group. Additionally, a simple logistic regression was performed to determine the predictive relationship between patient demographic variables and 30-day readmission, followed by a multiple regression model to determine which variables were significant predictors of readmission considering other covariates. Based on these results, the odds of 30-day readmission were greater for patients with longer initial hospital lengths of stay, with a previous hospitalization in the past 12 months, and in Black patients (compared to White patients) after adjusting for the following variables: insurance status, sex, age, hospital length of stay, hospital admission in previous 12 months, race, home antibiotic consultation, infection type (endocarditis, central nervous system, or skin or skin structure), chronic kidney disease, and heart failure (Table 4).

**Table 3.** Stratified Analysis of 30-Day Hospital Readmission

Variable	OPAT Management Program, No./Total (%)	Control, No./Total (%)	*P* Value^a^
Vancomycin use (n=116)	18/93 (19.4)	9/23 (39.1)	.0043
No vancomycin use (n=283)	31/150 (20.7)	21/133 (15.8)	.0228

Note. OPAT, outpatient parenteral antimicrobial therapy.

a χ^2^ test.

**Table 4.** Multiple Variable Logistic Regression Model of 30-day remission

Variable	Comparator (30-d readmission %, group total)	Reference (30-d readmission %, group total)	Adjusted Odds Ratio (95% CI)	*P* Value
Hospital length of stay	Mean=10.1	Any +1 day	1.04 (1.01–1.07)	.004
Hospital admission in past 12 mo	Yes (24.4%, n=201)	No (14.7%, n=191)	1.89 (1.09–3.28)	.023
Cefepime use	Yes (53.9%, n=26)	No (18.0%, n=373)	5.21 (2.14–12.64)	.003
Race	Black (41.5%, n=53)	White (16.0%, n=294)	4.18 (2.14–8.14)	.001
	Others (19.2%, n=52)	White (16.0%, n=294)	1.46 (0.66–3.24)	.403

Note. CI, confidence interval.

In total, 176 pharmacist interventions were made for patients in the OPAT management program. Moreover, 34.7% of the interventions were made once the patient was at home, and these primarily consisted of vancomycin dose adjustments. Other recommendations included dose adjustment at discharge (15.9%), management of adverse drug reactions (8.5%), advising a patient to seek medical attention (2.8%), assisting a patient in obtaining outpatient antibiotics (3.4%), or additional recommendations (34.7%) such as altering antimicrobial coverage when culture results were obtained after discharge, or changing antibiotic regimens for patient convenience or based on insurance coverage.

## Discussion

Although no statistically significant differences were detected between groups regarding the primary and secondary outcomes, these data do reveal a slight trend toward shorter hospital length of stay, shorter readmission length of stay, and reduced rates of infection-related readmission and ED visits in patients discharged with pharmacist-driven OPAT management. Given the retrospective nature of this study, the comparator groups were not well balanced regarding several baseline characteristics, including infection type, number of sites of infection, and OPAT duration and regimen. Rates of bone or joint infection were significantly higher among the OPAT cohort, which has the potential to skew the results of this study because these infections are difficult to cure and are associated with high rates of relapse and recurrence.^21^ Additionally, patients in the OPAT management program received OPAT for twice as long as the control group; thus, these patients were more likely to experience complications from extended course antibiotics or their PICC line. Patients in the OPAT management program were also more likely to have 2 or more sites of infection, resulting in more complicated antibiotic regimens. Given the magnitude of differences between study groups within this retrospective study, one could argue that the OPAT management program successfully maintained a similar readmission rate for a more complicated patient population compared to the control group. The post hoc analysis showed a significant difference in readmissions for patients receiving vancomycin, which may point to the value of pharmacist intervention for antimicrobials that require pharmacokinetic dosing and/or close safety monitoring.

There are major differences between our OPAT management program and other programs. Recently, Mansour et al^18^ reported a statistically significant reduction in 30-day readmission rates after implementing a nurse-managed OPAT program (20% before vs 13% after). This resulted in an estimated cost savings of $649,416 over 15 months. Although the nurse was responsible for performing a referral intake and patient counseling at hospital discharge, monitoring laboratory tests, and triaging calls from patients and nursing facilities, there was still a multidisciplinary approach including an overseeing ID physician, clinical pharmacists who were able to change doses (not agents), and clerical staff who assisted with phone calls. Additionally, only 50% of patients were receiving OPAT services from home, whereas the rest were discharged to skilled nursing facilities or dialysis centers.^18^ Another retrospective study on the outcomes of an OPAT service from a large teaching hospital in the United Kingdom demonstrated a readmission rate of only 7%, also using a multidisciplinary approach including ID physicians, specialist nurses, clerical staff, microbiology specialists, and clinical pharmacists.^2^ Our study only included patients who were discharged home with OPAT services, and they were communicated with solely via telephone. These patients did not have routine, follow-up clinic appointments to meet or speak with the pharmacist or the prescribing physician. This use of telemedicine to deliver OPAT is supported by the IDSA^22^ and UK good practice recommendations for OPAT.^17^ Additionally, the day-to-day operations of monitoring laboratory results; adjusting antibiotic doses; and communicating with patients, infusion companies, and home health providers were primarily pharmacist driven. This approach may be most suitable for environments with low resources available for OPAT monitoring, and in these circumstances, it may be most beneficial for patients at highest risk of complications or readmission.

Previous studies have identified risk factors for hospital readmission when receiving OPAT, including a history of diabetes, heart failure, renal failure, malignancy, or previous hospital admission in the last 12 months.^23,24^ These factors were collected in this study, and the odds of 30-day readmission were higher among patients with a hospital admission in the previous 12 months, in agreement with previous studies. Additionally, the odds of readmission were higher in patients with longer planned OPAT duration. Previous studies have also shown a higher rate of readmission in patients with bone and joint infections^25^ which was higher in the OPAT management program. Perhaps the most impactful finding was significant cost savings due to reduced hospital readmissions in patients on vancomycin receiving OPAT monitoring services. Because the OPAT service was often capped at our institution due to high consultation volume and lack of sufficient pharmacist manpower during the study period, these data were used to increase resources for the service.

This study had several limitations. First, the groups were not balanced at baseline because patients in the OPAT management program received more antibiotics, had longer durations of therapy, and had more sites of infection. The lack of randomization presents the likelihood that providers may have requested OPAT services for more complex patients. Since this was a retrospective chart review performed at a single institution, data could only be obtained regarding admissions and ED visits at our institution. We were unable to capture readmissions at outside institutions. Moreover, this patient population includes many who do not live in the metropolitan area, increasing the chances of patients seeking follow-up care elsewhere. Because some patients followed by the OPAT management program were advised by the pharmacist to seek medical attention at our ED, our rates of readmission or ED visits may have been falsely elevated compared to the control group because those patients may have otherwise sought care elsewhere. For example, patients within the OPAT management program had higher rates of readmission due to PICC-line complications and adverse drug reactions due to antibiotics (both of which may result in pharmacist-prompted ED visits) when compared with the control group. Patients in the control group had higher rates of readmission related to the index infection. Additionally, there may have been a lack of power to detect any differences in the primary and secondary outcomes based on the a priori power calculation. Lastly, all patients receiving vancomycin in the study were dosed using a trough-based dosing strategy instead of area under the curve, as recommended by recent guidelines, which has been shown to reduce rates of acute kidney injury (AKI) and possibly reduce readmissions.^26^ The study was also performed before daptomycin became available as a generic drug. Recent increased availability and convenience of outpatient daptomycin has led to decreased vancomycin utilization, thus reducing the need for pharmacokinetic monitoring.^27^

In conclusion, our findings confirm that patients treated via OPAT are risk of readmission within 30 days of discharge; patients in both groups had readmission rates ∼20%. Patients receiving vancomycin may benefit the most from home antibiotic monitoring, especially when trough-based dosing is used, as evidenced by the lower readmission rates and cost savings. Overall, in an environment with limited resources for OPAT, no difference was found in readmission rates or ED visits in patients followed by an OPAT management program provided via telephone. However, in similar institutions with low resources available for OPAT, a multidisciplinary OPAT management program may serve as an effective method to provide OPAT management to patients in greatest need or at highest risk for readmission, namely those with extended planned OPAT duration, with hospital admission in the previous 12 months, and with vancomycin-based OPAT regimens.
