# Impact of a Pharmacist-Managed Outpatient Parenteral Antimicrobial Therapy (OPAT) Service on Cost Savings and Clinical Outcomes at an Academic Medical Center – ADDENDUM

**DOI:** 10.1017/ash.2023.524

**Published:** 2023-12-18

**Authors:** Taylor M. Epperson, Kiya K. Bennett, Katherine K. Kupiec, Kathy Speigel, Stephen B. Neely, Beth H. Resman-Targoff, Karen K. Kinney, Bryan P. White

**Affiliations:** 1 Department of Pharmacy, Parkland Health, Dallas, Texas; 2 Clinical and Administrative Sciences, Department of Pharmacy, University of Oklahoma College of Pharmacy, Oklahoma City, Oklahoma; 3 Department of Pharmacy, University of Oklahoma Medical Center, Oklahoma City, Oklahoma; 4 Department of Nursing, University of Oklahoma Medical Center, Oklahoma City, Oklahoma; 5 Infectious Diseases Section, Department of Internal Medicine, University of Oklahoma College of Medicine, Oklahoma City, Oklahoma

In the initial publication of this article^1^, the following acknowledgements were omitted:

Jennifer Walling, PharmD, and Charles Whitman, PharmD, assisted in data collection.

The authors apologize for the omission.
